# Feasibility Study of a Smartphone Application for Detecting Skin Cancers in People With Albinism

**DOI:** 10.1200/GO.20.00264

**Published:** 2020-09-09

**Authors:** Fidel Rubagumya, Sarah K. Nyagabona, Ahuka N. Longombe, Achille Manirakiza, John Ngowi, Theoneste Maniragaba, Doriane Sabushimike, Sandra Urusaro, Diane A. Ndoli, Nazima Dharsee, Julius Mwaiselage, Daudi Mavura, Timothy P. Hanna, Nazik Hammad

**Affiliations:** ^1^ Department of Oncology, Rwanda Military Hospital, Kigali, Rwanda; ^2^ University of Global Health Equity, Burera, Rwanda; ^3^ Department of Epidemiolgy, Muhimbili University of Health and Allied Sciences, Dar es Salaam, Tanzania; ^4^ Polyclinique du Millénaire de Kisangani, Kisangani, Democratic Republic of Congo; ^5^ Department of Oncology, Ocean Road Cancer Institute, Dar es Salaam, Tanzania; ^6^ Department of Dermatology, Regional Dermatology Training Center, Moshi, Tanzania; ^7^ Kilimanjaro Christian Medical Center, Moshi, Tanzania; ^8^ Inshuti Mubuzima, Kigali, Rwanda; ^9^ Department of Oncology, Muhimbili University of Health and Allied Sciences, Dar es Salaam, Tanzania; ^10^ Division of Cancer Care and Epidemiology, Cancer Research Institute at Queen's University, Kingston, Canada; ^11^ Department of Oncology, Queen’s University, Kingston, Ontario, Canada

## Abstract

**PURPOSE:**

Albinism affects some facets of the eye’s function and coloration, as well as hair and skin color. The prevalence of albinism is estimated to be one in 2,000-5,000 people in sub-Saharan Africa and one in 270 in Tanzania. People in Tanzania with albinism experience sociocultural and economic disparities. Because of stigma related to albinism, they present to hospitals with advanced disease, including skin cancers. Mobile health (mHealth) can help to bridge some of the gaps in detection and treatment of skin cancers affecting this population.

**METHODS:**

We assessed the feasibility of using a mobile application (app) for detection of skin cancers among people with albinism. The study was approved by the Ocean Road Cancer Institute institutional review board. Data, including pictures of the lesions, were collected using a mobile smartphone and submitted to expert reviewers. Expert reviewers’ diagnosis options were benign, malignant, or unevaluable.

**RESULTS:**

A total of 77 lesions from different body locations of 69 participants were captured by the NgoziYangu mobile app. Sixty-two lesions (81%) were considered malignant via the app and referred for biopsy and histologic diagnosis. Of those referred, 55 lesions (89%) were biopsied, and 47 lesions (85%) were confirmed as skin malignancies, whereas eight (15%) were benign.

**CONCLUSION:**

With an increasing Internet coverage in Africa, there is potential for smartphone apps to improve health care delivery channels. It is important that mobile apps like NgoziYangu be explored to reduce diagnostic delay and improve the accuracy of detection of skin cancer, especially in stigmatized groups.

## INTRODUCTION

The risk of developing skin malignancies in the African population with albinism is estimated to be 1,000-fold higher than in the general population, with skin squamous cell carcinoma of the head and neck region being the most prevalent malignancy.^1,2^ In Tanzania, persons living with albinism experience economic disparities and social marginalization throughout their lives. Given the lack of formal education, they tend to have low-paying jobs that expose them to the sun, with poor-to-nonexistent skin protection measures.^3^ In addition, when diagnosed with skin cancers, they often have poor access to health care.

**CONTEXT**

**Key Objective**
To describe the feasibility of using a smartphone mobile health care (mHealth) application to aid in the detection of skin cancer in people with albinism. This application could be used to decrease health disparities for people with albinism by improving access to specialist care.
**Knowledge Generated**
Our study demonstrated that a smartphone application, NgoziYangu, can be used to aid the detection of skin cancers among people with albinism. We found use of this mHealth application feasible and worthy of additional investigation in resource-constrained settings.
**Relevance**
Tanzania is one of the African countries with a high number of people with albinism. They are faced with several skin diseases, including skin cancer. They are often subject to stigmatization, poverty, and brutal attacks, including mutilation, violence, and death. These create barriers to travel for specialist care. The use of mHealth could enhance access to care and decrease health disparities and inequities.

Albinism is a genetic condition caused by a complete lack of, or reduction in, melanin production.^4^ There are two major types of albinism: ocular albinism, affecting some facets of the eye’s function and coloration, and the most common, oculocutaneous albinism (OCA), which also affects hair and skin color.^4-6^ The latter is autosomal recessive, with seven subtypes. The most common albinism disorder in Equatorial Africa is OCA, and of interest in our context is subtype 2 (OCA-2). OCA-2 results from a mutation of the *OCA2* gene, which encodes the melanocyte-specific transporter protein.^7^

Although the number of detailed epidemiologic studies is limited, the prevalence of albinism is estimated to be one in 2,000-5,000 in sub-Saharan Africa.^8^ In Tanzania, the Tanzania Albinism Society (TAS) estimates that albinism likely affects one in 270 people or more than 150,000 individuals out of approximately 56 million nationally.^9^

To understand the natural history of skin cancer among people with albinism, in the 1980s, a cohort of people with albinism were enrolled in a study in Tanzania and followed from birth. They were found to have distinct skin changes during their first year of life, and two decades later, they were reported to develop considerable malignant changes with associated mortality in some cases.^10^ In an effort to curb late-stage presentations of skin cancers among people with albinism, this study and others made several recommendations for skin cancer prevention, ranging from community destigmatization and patient education to effective genetic counseling.^11^

Health-care-system– and patient-related delays have been reported for skin malignancy conditions, and variations exist, depending on the population’s level of awareness.^12^ Across low- and middle-income countries, access to cancer care is limited, and patient delays often reflect a lack of awareness, difficulties in access, and limited availability of health and diagnostic facilities.^13,14^ Even when care is available, patients, particularly those with albinism, often do not seek care because of stigma, which further delays or prevents diagnosis and treatment.^15^

Mobile health (mHealth), or the practice of medicine and public health supported by mobile devices, can help to bridge some of the gaps in both detection and treatment.^16^ One report estimates that mobile telecommunication technology use has reached nearly 46% in sub-Saharan Africa.^17^ Mobile phone applications (apps) assessing skin lesions in the general population, especially in high-income countries, have been used with various levels of success.^18-20^ Store-and-forward teledermatology has been reported to decrease delays in management of skin cancers, reduce referrals to a dermatologist, and limit treatment abandonment or loss to follow-up.^21^ However, little is known about such mHealth tools for skin cancer in low-income countries, particularly in people with albinism in Africa. Hence, the purpose of this study was to explore the feasibility of using a mobile app for detection of skin cancers in people with albinism in Tanzania.

## METHODS

This was a prospective study aimed at assessing the feasibility of using a mobile app for detection of skin cancers among people with albinism presenting with skin lesions in Dar es Salaam, Tanzania.

### The Mobile App and Data Entry

The mobile app, NgoziYangu (a Swahili word that means my skin), has functions to acquire images either directly through the mobile camera or by uploading an image that had already been taken and stored. It also allowed our research assistant to add patient information (eg, sex, contacts, date of birth, and so on) and skin lesion characteristics. The mobile device used for image acquisition was an Android smartphone with a 13-megapixel autofocus camera (1080 p and 30 fps per second in video mode).

Using the web app, our research assistant was able to enter the patients’ information and also upload images taken and stored earlier. The main purpose of the web app was for expert reviewers (an oncologist, a dermatologist, and a pathologist) to use their personal computers to view de-identified images and brief clinical information, and provide advice on management.

### Outcomes

We evaluated the proportion of patients flagged by the NgoziYangu app who were histologically confirmed to have cancer. Sensitivity, specificity, accuracy, and positive predictive value could not be directly measured because premalignant lesions and those deemed to be benign were not biopsied due to lack of funds.

### Data Collection

**FIG 1:**
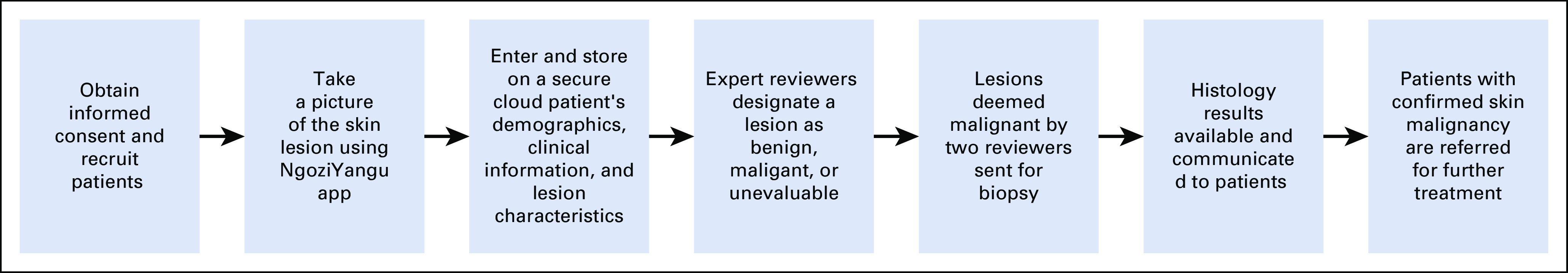
The process of patient recruitment to final disposition.

This study was approved by the Ocean Road Cancer Institute (ORCI) institutional review board. To participate in the study, patients received explanations about the study and signed informed consents. In conjunction with this study, ORCI started a screening program for people with albinism. The TAS made calls to its members with skin lesions inviting them to attend these clinics. The clinicians in these clinics were different from those evaluating the images remotely but were aware of our study. Our research assistant attended these clinics and collected data from patients who were screened. As illustrated in Figure 1, during these clinics, participants’ identification information (age, place of residence, occupation, and so on), lesion characteristics (site, duration of the lesion, number of lesions), and the image of the lesion were all captured. All the captured information and images were de-identified, securely stored on a cloud drive, and accessed by the expert reviewers. Expert reviewers’ diagnosis options were benign, malignant, or unevaluable (which required a picture to be retaken). Lesions deemed to be nonmalignant by two or all three reviewers were scheduled to be followed up every 3 months by TAS because the duration of the study was short and funds were limited. Patients with lesions deemed suggestive of malignancy by at least two of the three reviewers were referred for biopsies, and samples were sent for histologic review and reporting. Patients with pathologically confirmed biopsies were advised by the clinical team to receive additional treatment at ORCI. As a stakeholder in this study, TAS was charged with offering patient navigation services until the last disposition.

### Analytic Plan

The statistical analyses were performed with IBM SPSS Statistics version 22.0 (IBM, Armonk, NY). Because biopsies were not obtained from premalignant lesions or lesions clinically diagnosed as benign, test accuracy could not be determined. However, we performed a sensitivity analysis using available data to determine a range of possible accuracy, sensitivity, and specificity for the app using MedCalc. For this sensitivity analysis, we considered scenarios in which all benign and premalignant lesions were either (1) all pathologically proven malignancies or (2) all nonmalignant. We also considered a median scenario in which (3) half of all unbiopsied patients were malignant.

## RESULTS

The statistical analysis included all patients who were seen in the clinic for people with albinism at ORCI during the study period. A total of 77 lesions from different body locations in 69 participants were captured by the NgoziYangu mobile app (Table 1). The youngest patient with a skin lesion was 22 years of age, and the oldest was 67 years (median, 45 years), with a mean age of 47 years (standard deviation ± 10.98 years); 43 patients (62%) were males. The expert reviewers (a dermatologist, an oncologist, and a pathologist) considered 62 lesions (81%) to be malignant; therefore, patients with these lesions were referred for biopsy and histologic diagnosis (gold standard). Fifteen of 77 lesions (19%) were considered benign via the app. Five lesions were considered unevaluable by the reviewers, the photos were retaken, and they were counted among the 77. Of those referred, seven lesions (11%) were considered premalignant/benign and treated as such with cryotherapy, curettage, and/or ointments (imiquimod cream), whereas 55 lesions (89%) were biopsied. Among those biopsied, 47 lesions (85%) were confirmed as skin malignancies, whereas the remaining eight (15%) were benign. Among the patients with malignant disease, 31 (56%) had squamous cell carcinoma, 11 of whom (20%) had basal cell carcinoma and five (9%) had mixed type. Among the benign lesions, the most common diagnoses were actinic keratosis, acanthosis, solar elastosis, and orthokeratosis.

**TABLE 1:**
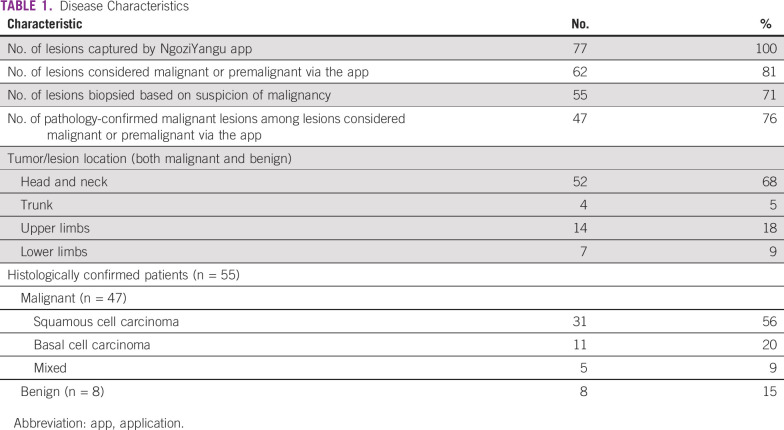
Disease Characteristics

## DISCUSSION

Tanzania is believed to be one of the African countries with a high number of people with albinism.^22^ People with albinism in Tanzania and sub-Saharan Africa in general are faced with several health inequities and disparities.^23^ This vulnerable group is often subject to stigmatization and brutal attacks, including mutilation, violence, and death.^24^ They also are prone to various types of skin lesions and skin cancers. Most are peasant farmers and others have low-paying jobs with no proper source of income to facilitate their transport to seek and pay for medical care. Most present with late stages of skin cancer.^25^ Persistent stigmatization, discrimination, and persecution compound poor access to health care, resulting in poor health outcomes.^26^

With the scarcity of dermatologic services and trained dermatologists in Tanzania, innovative approaches involving the use of mobile apps could decrease the number of people with albinism whose skin lesions would otherwise be left unattended, consequently with delayed cancer diagnosis.^27^

To our knowledge, the NgoziYangu app was the first teledermatology mobile app in Tanzania and the first in sub-Saharan Africa used for early detection of skin cancers in a population of people with albinism. Additionally, most dermato-oncology mobile apps were designed for melanoma, whereas the NgoziYangu app targeted all potentially malignant skin lesions affecting people with albinism. In contrast to other mobile apps that use automated analysis, which has a high likelihood of giving false-negative results, the NgoziYangu app uses a store-and-forward image-based system, which has been shown to have better accuracy.^28^ In this study, 76% of lesions considered malignant or premalignant via the app were pathologically proven to be cancer. Automated image analysis by a smartphone algorithm for melanoma has reported 80% accuracy to detect cancer.^20^ Although the accuracy of NgoziYangu could not be determined because of the limitations of this study, our sensitivity analysis suggests the accuracy of the app is likely to be favorable (Table 2).

**TABLE 2:**
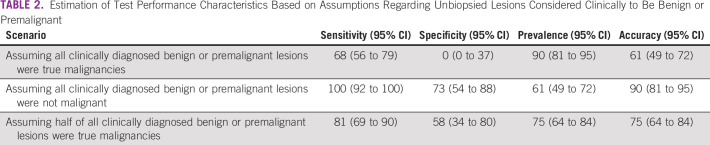
Estimation of Test Performance Characteristics Based on Assumptions Regarding Unbiopsied Lesions Considered Clinically to Be Benign or Premalignant

Despite its large size and population, Tanzania has an Internet penetration rate of 46%^29^ and has witnessed an increasing uptake of mobile technology by the poor or remote populations. The use of mHealth will be the most convenient, fastest, and cost-effective way to connect specialized care that would otherwise be unreachable to vulnerable groups like people living with albinism.

Our study suggests that combined with other enabling factors, including the assistance of the TAS, a coordinated care plan, and availability of the Internet, people with albinism presenting with skin lesions could possibly undergo an initial evaluation remotely using mobile apps embedded in smartphones. Patients with lesions suggestive of malignancy can be carefully linked to the point of care without unnecessary referral. Given the low number of people with albinism attending the ORCI clinic, we expect that such an approach could have a major impact on minimizing diagnostic delay and ensuring presentation to health providers. Challenges include the distance from Dar es Salaam to Kilimanjaro Medical Center, which is located in the Moshi region (338 miles away). This can be mitigated by equipping regional referral hospitals to take care of basic needs, including biopsy.

This study also demonstrated the feasibility of collaboration between academic centers and advocacy organizations, such as the TAS. This partnership allowed delivery of care and research that hopefully will help decrease the equity gap experienced by people living with Albinism.

Although the use of smartphone apps in health care offers hope for improved quality of care, it also raises several ethical and legal concerns, such as data security and confidentiality.^30^ Teledermatology needs to be supplemented with other clinical information, because in many instances, photographs alone are not enough.^31^ Notably, in our care process, a clinician evaluated the skin lesion in person, and benign lesions were treated without biopsy. Mobile apps should not replace standard health care providers’ consultations because diagnostic inaccuracy is possible.^18^

Limitations of this study included not being able to biopsy lesions that were deemed to be benign by the NgoziYangu app. This could have helped test the accuracy of the app. However, we performed a sensitivity analysis that suggested that the app is likely to have reasonable accuracy and sensitivity. Field testing of the app between ORCI and remote sites is also required. The acceptability of the technology in the community of people with albinism and the impact on mitigating diagnostic delay also need to be determined.

Technologies that harness the increasing Internet coverage in Africa such as NgoziYangu have the potential to enhance access to care and decrease disparities for people with albinism by linking vulnerable patients with health care professionals. Our study has demonstrated that use of such technology is feasible and worthy of additional investigation. Future studies should ascertain the safety, accuracy, acceptability, and impact on health outcomes of such mobile apps, particularly in people with albinism.
